# Proceedings: The carcinogenicity of oil mist.

**DOI:** 10.1038/bjc.1975.203

**Published:** 1975-08

**Authors:** H. A. Waldron


**THE CARCINOGENICITY OF OIL MIST.** H. A. WALDRON, Department of Social Medicine, The Medical School, Birmingham.

There is no doubt that exposure to mineral oil may induce skin tumours in susceptible individuals but there is some controversy regarding the carcinogenicity of oil mist (*Lancet*, 1970 ii, 967). A preliminary study of a series of men with a first primary tumour of the scrotum revealed an excess of subsequent primary tumours, notably of the bronchus and digestive tract. (Holmes, Kipling and Waterhouse, *Lancet*, 1970, ii, 214; Waterhouse, *Ann. occup. Hyg.*, 1971, **14**, 161; Waterhouse, *Ann. occup. Hyg.*, 1972, **15**, 43). The present study has extended these findings, particularly as they relate to exposure to oil mist.

A total of 288 cases of scrotal cancer was registered at the Birmingham Regional Cancer Registry (BRCR) between 1936 and 1971 and in this group of men, 42 subsequent primary tumours were registered between 1936 and 1972 (allowing for at least a one year follow-up period). The expected number of second primary tumours was 17.11 ($P < 0{\cdot}001$). Analysis of the sites of the second primary tumours showed a significant excess in the larynx, bronchus, lip, stomach and skin. An examination was made of the occupations of the cases and these were divided into 4 sub-groups, those with exposure to oil (162) those with exposure to pitch and tar (36), those in whom exposure to known carcinogens was uncertain (73) and those whose occupation was unknown (17). With 2 exceptions, the excess of second primary tumours was confined to the group with oil exposure (see Table I). The exceptions were that an excess of skin tumours was found in the men with exposure to pitch and tar (3 observed, 0·05 expected, $P < 0·001$) and an excess of tumours of the stomach was noted in the men whose exposure was uncertain (3 observed, 0·59 expected, $P < 0·05$). The first excess is to be anticipated on *a priori* grounds, but no explanation can be offered for the excess of stomach tumours in the absence of knowledge of exposure to possible carcinogens.

The finding of an excess number of second primary tumours of the larynx and bronchus in the oil exposed group is consistent with the notion that oil mist is acting as a carcinogen. To test this hypothesis, the occupations of all the male cases of carcinoma of the bronchus and larynx registered at the BRCR between 1967 and 1969 were catetorized according to the Registrar General's classification of occupation. The proportion of men in each of the occupational orders was compared with the number of men in each order as a proportion of the total work force in the region during the same years, the assumption being that if tumours of these sites were related to oil exposure, then a significant excess should be observed in those orders which included men in oily jobs (order V and VII). An overall excess of bronchial tumours was noted in order V ($P < 0·001$) but not in order VII. Neither order showed an excess of laryngeal tumours. The excess of bronchial tumours in order V was due to an excess in 2 categories of workers, metal furnace men, and smiths and forgemen.

The 2 occupations which between them contributed over half the cases of second primary tumours in the index series were the toolmakers and machine operators. In the regional analysis, machine operators were represented according to expectation, but the toolmakers showed a significant *deficit* of both bronchial and laryngeal tumours ($P <$ 0·001 and $< 0·05$ respectively).

TABLE I

| Site | Expected number | Observed number | $P$ |
|---|---|---|---|
| All sites | 8·24 | 28 | $<0·001$ |
| Larynx | 0·13 | 2 | $<0·05$ |
| Bronchus | 2·64 | 11 | $<0·001$ |
| Skin | 0·77 | 11 | $<0·001$ |
| Remainder | 4·70 | 4 | n.s. |

Expected and observed number of subsequent primary tumours in men with oil exposure.

Thus, if oil mist is carcinogenic, this effect appears to be exerted on only a sub-set of the exposed population composed, presumably, of persons having an enhanced susceptibility, the basis of which is unclear.


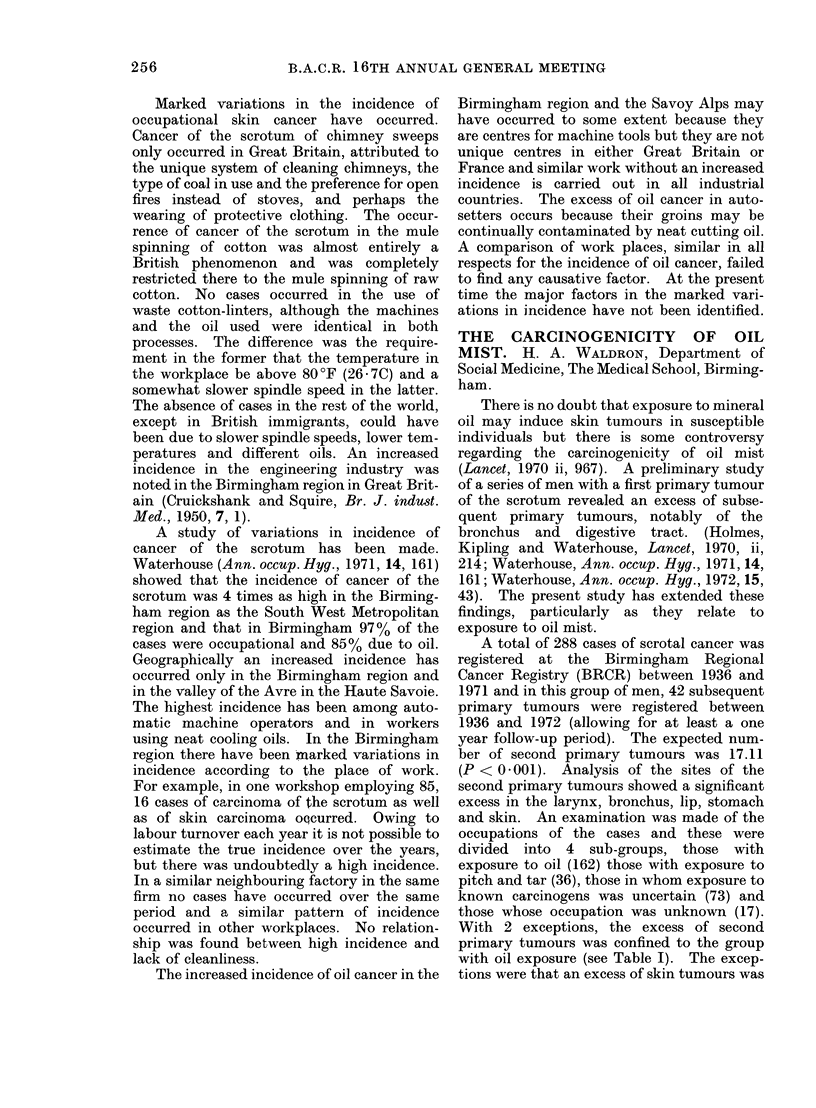

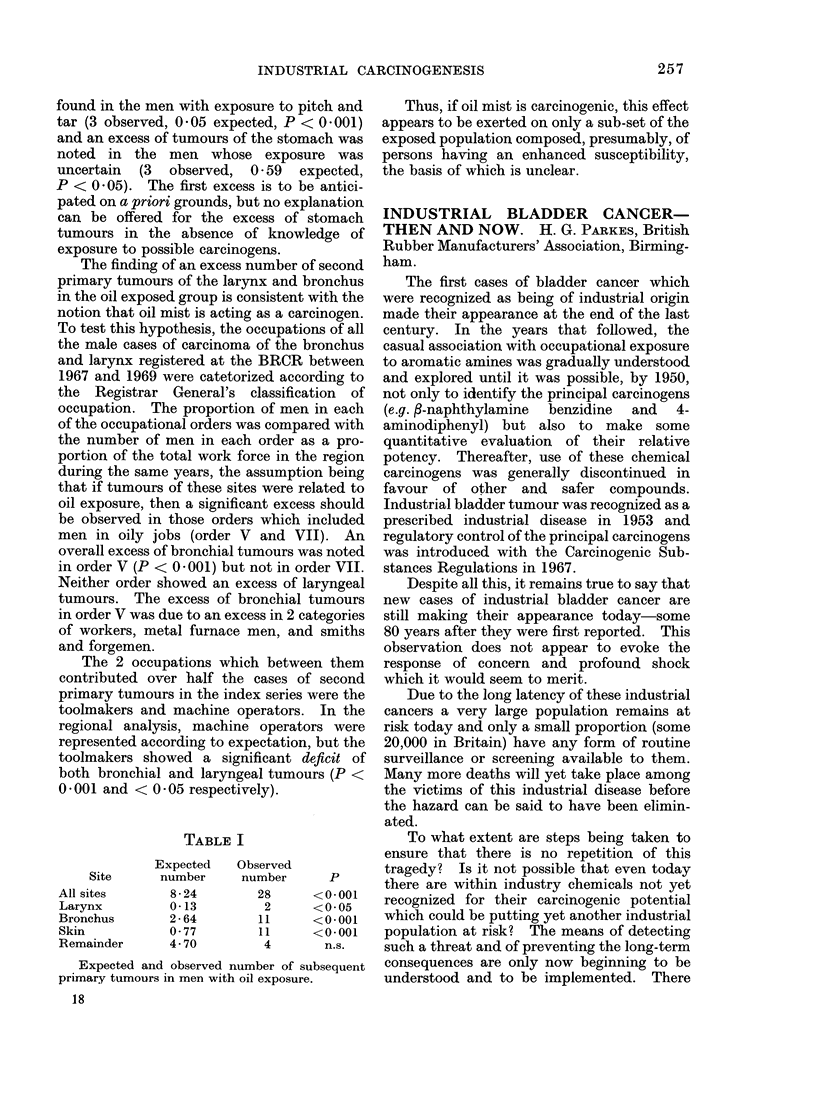


## References

[OCR_00018] Waterhouse J. A. (1971). Cutting oils and cancer.. Ann Occup Hyg.

